# Oxidative Stress-Driven Cellular Senescence: Mechanistic Crosstalk and Therapeutic Horizons

**DOI:** 10.3390/antiox14080987

**Published:** 2025-08-12

**Authors:** Bojan Stojanovic, Ivan Jovanovic, Milica Dimitrijevic Stojanovic, Bojana S. Stojanovic, Vojin Kovacevic, Ivan Radosavljevic, Danijela Jovanovic, Marina Miletic Kovacevic, Nenad Zornic, Ana Azanjac Arsic, Stevan Eric, Nikola Mirkovic, Jelena Nesic, Stefan Jakovljevic, Snezana Lazarevic, Ivana Milivojcevic Bevc, Bojan Milosevic

**Affiliations:** 1 Department of Surgery, Faculty of Medical Sciences, University of Kragujevac, 34000 Kragujevac, Serbia; bojan.stojanovic01@gmail.com (B.S.);; 2 Center for Molecular Medicine and Stem Cell Research, Faculty of Medical Sciences, University of Kragujevac, 34000 Kragujevac, Serbia; 3 Department of Pathology, Faculty of Medical Sciences, University of Kragujevac, 34000 Kragujevac, Serbia; 4 Department of Pathophysiology, Faculty of Medical Sciences, University of Kragujevac, 34000 Kragujevac, Serbia; 5 Department of Histology and Embryology, Faculty of Medical Sciences, University of Kragujevac, 34000 Kragujevac, Serbia; 6 Department of Neurology, Faculty of Medical Sciences, University of Kragujevac, 34000 Kragujevac, Serbia; 7 Department of Internal Medicine, Faculty of Medical Sciences, University of Kragujevac, 34000 Kragujevac, Serbia; 8 City Medical Emergency Department, 11000 Belgrade, Serbia

**Keywords:** oxidativestress, cellular senescence, mitochondrial dysfunction, SASP, redox signaling, DNA damage response, Nrf2 pathway, senotherapy, aging, cancer

## Abstract

Cellular senescence, a state of permanent cell cycle arrest, represents a double-edged sword in biology—providing tumor-suppressive functions while contributing to tissue degeneration, chronic inflammation, and age-related diseases when senescent cells persist. A key driver of senescence is oxidative stress, primarily mediated by excessive reactive oxygen species that damage mitochondrial DNA, modulate redox-sensitive signaling pathways, and trigger the senescence-associated secretory phenotype. Emerging evidence highlights the pathogenic role of SASP in promoting local inflammation, immune evasion, and senescence propagation. This review explores the intricate interplay between redox imbalance and cellular senescence, emphasizing mitochondrial dysfunction, SASP dynamics, and their implications in aging and cancer. We discuss current senotherapeutic strategies—including senolytics, senomorphics, antioxidants, gene therapy, and immunotherapy—that aim to eliminate or modulate senescent cells to restore tissue homeostasis. Understanding the heterogeneity and context-specific behavior of senescent cells remains crucial for optimizing these therapies. Future research should focus on addressing key knowledge gaps, including the standardization of senescence biomarkers such as circulating miRNAs, refinement of predictive preclinical models, and development of composite clinical endpoints. These efforts are essential to translate mechanistic insights into effective senotherapeutic interventions and enable the safe integration of senescence-targeting strategies into routine clinical practice.

## 1. Introduction

Cellular senescence, a stable and irreversible form of cell cycle arrest, has emerged as a central biological process at the intersection of aging, chronic diseases, and cancer [1]. While initially recognized for its tumor-suppressive role by preventing the proliferation of damaged cells, senescence is now understood as a complex, context-dependent phenomenon that also drives tissue dysfunction, chronic inflammation, and age-related pathologies when senescent cells accumulate [2]. A pivotal factor in the induction and maintenance of senescence is oxidative stress, driven by an imbalance between reactive oxygen species (ROS) production and antioxidant defenses [3]. Excessive ROS not only cause direct macromolecular damage but also initiate signaling cascades that reinforce senescence and trigger the senescence-associated secretory phenotype (SASP), a pro-inflammatory state with systemic implications [3]. Recent studies emphasize the complex interaction between redox imbalance, mitochondrial dysfunction, deoxyribonucleic acid (DNA)damage response, and changes in cellular metabolism that help to maintain senescence. However, there is still debate about the double-edged role of senescence in both health and disease, as well as the most effective ways to target it therapeutically. In this comprehensive review, we explore the mechanistic links between oxidative stress and cellular senescence, summarize emerging senotherapeutic approaches—including senolytics, senomorphics, gene therapies, and redox modulators—and discuss the translational challenges in targeting senescence to improve healthspan and manage age-related diseases.

## 2. Definition and Biological Nature of Reactive Oxygen Species

The concept of oxidative stress was first explored in the mid-20th century and has gained increasing scientific interest since the 1970s [4]. It was originally described as an imbalance between oxidants and antioxidants, favoring the oxidants and leading to potential cellular damage [5]. Later, this concept was expanded to include disruption in redox signaling and regulation [6].

Reactive oxygen species are chemically reactive molecules that contain oxygen [7]. They include both radical forms—such as superoxide anion (O_2_^−^•) and hydroxyl radical (OH•)—and non-radical forms like hydrogen peroxide (H_2_O_2_) [7]. Some of these species are stable, while others are highly reactive and capable of causing significant cellular damage [7]. ROS can also interact with reactive nitrogen species (RNS), such as nitric oxide (NO) and peroxynitrite (ONOO^−^), leading to similar harmful effects [8,9].

Among various ROS, those classified as highly reactive (hiROS), such as hydroxyl radicals and singlet oxygen, are especially damaging to proteins, lipids, and DNA [10]. Less reactive species (loROS), such as superoxide and hydrogen peroxide, are often involved in physiological processes like intracellular signaling [10].

This entire network of ROS, sometimes referred to as the ROS physiome, includes a wide variety of molecules with different reactivities [11]. While some ROS contribute to important cellular functions, excessive accumulation leads to oxidative stress and may initiate pathological processes [11].

### 2.1. Endogenous and Exogenous Sources of Reactive Oxygen and Nitrogen Species

Reactive oxygen species and reactive nitrogen species are continuously generated in biological systems, both under normal physiological conditions and in response to pathological stimuli [11,12]. The mitochondria are widely recognized as a major source of intracellular ROS, primarily through electron leakage during oxidative phosphorylation [13]. Inefficient electron transfer at respiratory complexes I and III leads to partial reduction of oxygen, forming superoxide anions and subsequently other ROS [14]. Given that adenosine triphosphate (ATP) synthesis is tightly coupled with oxygen consumption, mitochondrial metabolism inherently generates ROS as a byproduct of aerobic respiration [15].

In addition tomitochondrial respiration, ROS are also generated by several enzyme systems. These include nicotinamide adenine dinucleotide phosphate (NADPH) oxidases (NOX1–5), dual oxidases (DUOX1–2), xanthine oxidase, nitric oxide synthases, cytochrome P450 enzymes, and monoamine oxidases [16]. These enzymes are distributed across distinct cellular compartments such as the cytosol, plasma membrane, endoplasmic reticulum, and peroxisomes, each contributing to the oxidative burden in different contexts [17]. For example, peroxisomal β-oxidation of fatty acids and the activity of oxidoreductases also generate ROS, further amplifying redox signaling and oxidative damage [18].

Exogenous factors also play a substantial role in ROS/RNS generation [19]. Various external factors can trigger or increase the production of ROS. These include environmental pollutants, ionizing radiation, xenobiotics, certain drugs, such as chemotherapeutics, and specific dietary components [20,21,22]. Additionally, during immune responses, phagocytic cells such as neutrophils and macrophages produce a burst of ROS and RNS via NADPH oxidase and inducible nitric oxide synthase as part of host defense mechanisms [23,24].

### 2.2. Antioxidant Defense Networks and the Nrf2 Axis in Redox Homeostasis and Aging

The dynamic equilibrium between prooxidant and antioxidant forces governs cellular redox homeostasis and is vital for the preservation of physiological function [25]. Under basal conditions, this balance supports signaling fidelity, energy metabolism, and cellular integrity [26]. However, disruption of this equilibrium, typically in favor of oxidants, leads to oxidative and nitrosative stress, driving molecular damage and contributing to the onset of aging and degenerative diseases [26].

To mitigate oxidative insults, cells have evolved an intricate antioxidant defense system encompassing both enzymatic and non-enzymatic components [27]. These mechanisms function across three principal levels: preventing the formation of ROS, intercepting and neutralizing radicals, and repairing or removing oxidatively damaged biomolecules [28].

The frontline defense consists of enzymatic antioxidants, including superoxide dismutases (SODs), catalase (CAT), glutathione peroxidases (GPxs), glutathione reductase (GR), glutamyl transpeptidase (GGT), and thioredoxin reductases [29,30]. These enzymes neutralize ROS through catalysis, thereby preventing chain reactions that would otherwise propagate cellular injury [30]. Non-enzymatic antioxidants, such as reduced glutathione, ascorbate (vitamin C), tocopherol (vitamin E), uric acid, and carotenoids, act as radical scavengers by donating electrons, effectively terminating oxidative cascades without becoming reactive themselves [31,32].

Preventive antioxidants help to reduce the formation of ROS by binding transition metals like iron and copper. Plasma proteins such as transferrin, ferritin, ceruloplasmin, and albumin act in this way, limiting Fenton chemistry and preventing further ROS production [28,33]. A third layer of protection involves enzymatic repair systems that degrade oxidized lipids, proteins, and nucleic acids, alongside regeneration pathways that restore damaged macromolecules [28].

Central to redox regulation is the Kelch-like ECH-associated protein 1–nuclear factor erythroid 2–related factor 2 (Keap1–Nrf2) signaling pathway [34]. Under homeostatic conditions, Nrf2 is sequestered in the cytoplasm by Keap1, which targets it for ubiquitin-mediated degradation [34,35]. Upon oxidative or electrophilic challenge, conformational changes in Keap1 release Nrf2, allowing its nuclear translocation [36]. In the nucleus, Nrf2 forms a complex with small musculoaponeurotic fibrosarcoma (Maf) proteins. This complex binds to antioxidant response elements (AREs) and activates the transcription of several antioxidant genes. These include heme oxygenase-1 (HO-1), NAD(P)H quinone oxidoreductase 1 (NQO1), superoxide dismutases (SODs), glutathione peroxidases (GPxs), ferritin, and thioredoxin [36,37].

This transcriptional network coordinates an adaptive response to redox perturbations and is pivotal for cytoprotection. However, Nrf2 activity declines with age, rendering cells more susceptible to oxidative stress and promoting senescence. Conversely, pharmacological activation of Nrf2 has been shown to delay senescence and extend cellular lifespan in preclinical models [35].

Exogenous antioxidants derived from the diet complement endogenous systems [38]. These include trace elements (e.g., selenium, zinc), vitamins C and E, carotenoids, and a broad spectrum of phytochemicals found in fruits, vegetables, coffee, tea, and whole grains [28]. Such compounds reduce lipid peroxidation, modulate signaling cascades, and support enzymatic antioxidant activity. In populations with impaired endogenous defenses, such as the elderly or individuals with chronic disease, dietary antioxidants may provide significant protection [28]. Elevated plasma levels of vitamin C and β-carotene have been correlated with improved cognitive performance and reduced oxidative stress in aging cohorts [39].

### 2.3. Circadian Regulation of Nrf2 Signaling: A Bidirectional Link Between the Molecular Clock and Redox Homeostasis

Emerging evidence indicates that the Nrf2/ARE pathway is under circadian regulation, integrating redox homeostasis with the body’s internal clock [40]. This link is of particular relevance in the context of aging and senescence, as both redox imbalance and circadian disruption are known hallmarks of aging tissues [41].

The molecular circadian clock is driven by two interlocking transcriptional–translational feedback loops (TTFLs) [42]. In the primary loop, the heterodimer composed of Circadian Locomotor Output Cycles Kaput (CLOCK) and Brain and Muscle ARNT-Like 1 (BMAL1) activates the transcription of core clock genes by binding to E-box elements within their promoter regions [42]. These target genes include *Period* homologs (PER1, PER2, and PER3) and *Cryptochrome* homologs (CRY1 and CRY2), which function as negative regulators by inhibiting the activity of the CLOCK/BMAL1 complex, thereby generating circadian oscillations [42].

Importantly, BMAL1 has been shown to bind directly to E-box motifs in the promoter region of the *Nrf2* gene, thereby regulating the rhythmic expression of Nrf2 and its downstream antioxidant genes such as NAD(P)H quinone dehydrogenase 1 (NQO1), heme oxygenase 1 (HMOX1), and glutamate–cysteine ligase modifier subunit (GCLM) [43]. Cells show decreased antioxidant capacity and increased vulnerability to oxidative damage when Nrf2 expression reaches its lowest point during the circadian cycle [44].

BMAL1 thus plays a dual role as a regulator of both circadian rhythm and redox balance [45]. Studies in human lens epithelial cells have shown that aging is accompanied by a decline in BMAL1–CLOCK expression and a concomitant reduction in Nrf2-regulated antioxidant gene expression [46]. This is associated with increased levels of ROS. Overexpression of BMAL1 in these cells enhances the expression of Nrf2, NQO1, SOD, and peroxiredoxin 6 (PRDX6) at both the mRNA and protein levels, resulting in reduced oxidative stress [46].

BMAL1 can also activate nuclear factor Nrf2, which then interacts with other stress-response pathways. These include the nuclear factor kappa B (NF-κB) signaling pathway and the heat shock response (HSR). Together, they form a protective network that defends cells against ROS-induced apoptosis, inflammation, and tissue damage [40]. Loss of BMAL1 impairs this network: its deletion suppresses Nrf2 expression and antioxidant defense while simultaneously increasing the production of interleukin-1 beta (IL-1β), a proinflammatory cytokine [40].

Intriguingly, the relationship is bidirectional. Nrf2 can also influence circadian rhythm. In hepatocytes, activated Nrf2 has been shown to bind to enhancer regions of the *Cryptochrome 2* (CRY2) gene, enhancing its expression and thereby modulating CLOCK/BMAL1-driven transcription. These findings suggest that Nrf2 and the circadian clock form an integrated feedback loop that synchronizes redox signaling with tissue-specific circadian timekeeping [40].

### 2.4. Redox Signaling: Dual Role of ROS in Cell Fate and Disease Progression

Beyond their damaging potential, ROS are now widely recognized as integral regulators of diverse cellular processes [47]. These include cell signaling, gene regulation, immune system activity, and control of cell survival, growth, and programmed cell death [47,48]. The concept of “redox biology” has emerged to describe this functional dimension of ROS, highlighting their role as signaling intermediates rather than mere byproducts of metabolism [47].

The effects of ROS on cells depend on their concentration and the specific cellular context [49]. Low-to-moderate levels modulate redox-sensitive signaling cascades, whereas excessive or prolonged exposure shifts ROS from regulatory agents to cytotoxic mediators [31]. Their effects are dictated by molecular species, subcellular localization, and interaction with target proteins [50]. In particular, redox-sensitive transcription factors and kinases, such as Nrf2, tumor protein p53 (p53), NF-κB, forkhead box O (FOXO), activator protein 1 (AP-1), and hypoxia-inducible factor 1-alpha (HIF-1α), serve as redox sensors, initiating adaptive or pathological responses depending on ROS dynamics [3].

In stem cell biology, ROS act as molecular switches that translate external signals such as nutrients, cytokines, and oxygen levels into internal decisions that control self-renewal, dormancy, or differentiation [51,52]. However, sustained or excessive ROS levels can drive stem cell exhaustion and compromise regenerative potential [51]. Reactive oxygen species produced by mitochondria also play a key role in “mitohormesis”. This is a process where low levels of oxidative stress trigger beneficial adaptive responses, helping to extend healthspan and delay the development of age-related diseases [53].

On the other hand, chronic oxidative or nitrosative stress resulting from persistent ROS accumulation or impaired detoxification disturbs redox homeostasis [31]. This leads to lipid peroxidation, protein oxidation, and DNA damage, particularly within mitochondrial and nuclear genomes [31]. Inadequate DNA repair mechanisms under oxidative stress conditions contribute to genomic instability and disease susceptibility [54].

High ROS levels are hallmarks of several chronic disorders, including diabetes, atherosclerosis, neurodegeneration, and inflammatory diseases [55]. In immune cells such as neutrophils and macrophages, ROS are crucial for microbial killing [56]. However, sustained activation contributes to tissue damage and chronic inflammation [31]. In the context of cancer, ROS exert paradoxical effects;they can both promote tumor initiation via DNA damage and mutations and support tumor progression by modulating oncogenic pathways, evading apoptosis, and sustaining proliferative signaling [57,58]. The interplay between ROS, p53, and NF-κB is particularly significant in shaping tumor cell fate and metastatic behavior [59]. Furthermore, oncogene-driven metabolic reprogramming can amplify ROS production, reinforcing malignant phenotypes [59].

An integrated summary of ROS types, sources, antioxidant systems, and their physiological and pathological roles is provided in Table 1.

## 3. Cellular Senescence: A Double-Edged Regulator of Aging and Disease

Cellular senescence represents a stable and irreversible state of cell cycle arrest initiated by a variety of intrinsic and extrinsic stressors, including telomere attrition, DNA damage, oxidative stress, and oncogene activation [60]. Senescence was first observed in cultured fibroblasts, where cells lost the ability to divide after a certain number of cycles. Today, it is recognized as a wider biological process involved in development, tissue repair, cancer prevention, and aging [2].

Senescent cells show several structural and molecular changes. These include an increase in cell size, a flattened shape, and expression of senescence-associated β-galactosidase (SA-β-gal). They also activate the DNA damage response (DDR), form senescence-associated heterochromatin foci (SAHF), and release inflammatory factors known as the senescence-associated secretory phenotype (SASP) [1]. Central regulators of the senescence program include cyclin-dependent kinase inhibitors such as p16^INK4a^ and p21^CIP1^, which enforce proliferative arrest by inhibiting key cell cycle regulators [60].

Crucially, new data indicates that circadian regulation affects both p16 and p21 [45]. Time-of-day-dependent control of cell cycle progression is facilitated by the nuclear RNA-binding protein NONO (non-POU domain-containing octamer-binding protein), which has been identified as a circadian output effector that directly and rhythmically activates p16^INK4a^ transcription [61]. Similar to this, the circadian clock regulates the expression of p21^CIP1/WAF1^, and changes in core clock genes can change p21 levels, which can impact the fidelity and timing of cell cycle arrest [45].

Importantly, senescence can exert both beneficial and detrimental effects [62]. In its acute form, it is a well-regulated process that affects specific cell populations during tissue repair or after injury [2]. These senescent cells often promote their own removal by releasing signals through the SASP, which attracts immune cells and helps to restore tissue balance [2]. In contrast, chronic senescence arises from prolonged exposure to damaging stimuli and is marked by the persistence of senescent cells that escape immune clearance. These cells accumulate over time and contribute to the chronic inflammation and tissue dysfunction observed in aging and age-related diseases, including cancer, atherosclerosis, neurodegeneration, and renal pathologies [63].

### 3.1. Molecular Triggers of Cellular Senescence

Cellular senescence can be initiated by a variety of intrinsic and extrinsic stressors that converge on key signaling pathways to enforce a stable and irreversible cell cycle arrest [60]. These stimuli often reflect damage or dysfunction at the genomic, metabolic, or organelle level, serving as protective mechanisms to prevent propagation of potentially harmful cells [64]. However, the chronic accumulation of senescent cells contributes to tissue dysfunction and aging-related pathology [65]. The principal molecular triggers of senescence include telomere attrition, DNA damage, oxidative and metabolic stress, mitochondrial dysfunction, endoplasmic reticulum (ER) stress, and oncogenic signaling [2].

One of the earliest described forms of senescence, known as replicative senescence, arises from progressive telomere shortening [66]. Telomeres, composed of repetitive nucleotide sequences, protect chromosome ends from degradation [67]. Because of the limitations in DNA replication, known as the end-replication problem, telomeres become shorter with each cell division [68]. When telomeres become too short, the exposed DNA ends trigger a continuous DDR, which causes the cell to permanently stop dividing [66]. This checkpoint, triggered by telomere shortening, acts as a built-in timer that limits how many times a somatic cell can divide. It helps to protect the genome from instability [69,70].

Cells exposed to genotoxic agents—such as ultraviolet (UV) or ionizing radiation, oxidative byproducts of metabolism, or certain chemotherapeutic agents—develop DNA lesions that activate canonical DDR pathways [71]. This response is initiated by the sensing of DNA double-strand breaks (DSBs) primarily by the MRN complex (MRE11-RAD50-NBS1), which recruits and activates ATM (ataxia telangiectasia mutated) and ATR (ATM and Rad3-related) kinases [72].

ATM and ATR phosphorylate histone variant H2AX (forming γH2AX), which acts as a platform for the recruitment of DNA repair machinery and checkpoint signaling proteins such as CHK1 and CHK2 [73]. These kinases subsequently stabilize and activate the tumor suppressor p53, which induces the transcription of CDK inhibitor p21^CIP1/WAF1^. This inhibits CDK2 activity and prevents phosphorylation of the retinoblastoma protein (pRb), enforcing a G1/S cell cycle arrest [60,74].

Simultaneously, persistent stress or replicative signals upregulate p16^INK4a^, which inhibits CDK4/6 and reinforces the hypophosphorylated state of pRb [60]. The cooperation between the p53/p21 and p16/pRb pathways is essential for establishing and maintaining stable growth arrest characteristic of senescent cells [60]. Importantly, while p21 mediates the initial, reversible arrest in response to acute damage, p16 is associated with long-term, irreversible senescence, particularly in the context of telomere dysfunction and chronic oxidative stress [60]. These mechanisms together represent a protective, tumor-suppressive barrier that prevents propagation of cells with genomic instability.

Mitochondria are central regulators of energy metabolism and redox balance [75]. Dysfunction of the electron transport chain (ETC), especially at complexes I and III, leads to electron leakage and excessive ROS production [75]. These ROS can damage mitochondrial and nuclear components, inducing senescence through p53 and p21 activation [76]. Senescent cells triggered by mitochondrial impairment often shift from oxidative phosphorylation to glycolysis (Warburg-like metabolism), exhibit altered mitochondrial dynamics, and display persistent SASP expression [77]. The interplay between mitochondrial ROS and senescence establishes a self-perpetuating cycle of damage, metabolic reprogramming, and inflammatory signaling that contributes to tissue aging [62].

Metabolic stress, induced by conditions such as glucose overload or nutrient deprivation, contributes significantly to the induction and maintenance of cellular senescence [78]. Senescent cells experience major changes in their metabolism. They rely more on glycolysis, show reduced mitochondrial oxidative phosphorylation, and accumulate damaged mitochondria that produce high levels of ROS [79]. These metabolic changes contribute to redox imbalance and help to maintain the senescent state. They are regulated by nutrient and energy sensing pathways, particularly mTOR and AMPK signaling networks [62]. Chronic activation of mTOR signaling enhances anabolic metabolism, increases oxidative stress, and promotes the synthesis of SASP factors through the IL-1α–NF-κB axis [80]. In contrast, AMPK responds to energy stress by inhibiting mTOR activity and promoting autophagy and catabolic pathways, which help to mitigate oxidative burden and delay the onset of senescence [81]. The tumor suppressor protein p53 also contributes to the metabolic regulation of senescence by modulating mitochondrial function, glycolysis, and the expression of antioxidant enzymes. Experimental evidence from model organisms indicates that interventions targeting these metabolic pathways, such as caloric restriction or pharmacological mTOR inhibition, can restore metabolic homeostasis, suppress SASP expression, and extend both healthspan and lifespan [82].

The endoplasmic reticulum (ER) plays a central role in protein folding, post-translational modification, and quality control, ensuring proper proteostasis within the cell [83]. When the ER’s folding capacity is overwhelmed, due to excessive protein synthesis, oxidative stress, or perturbations in calcium homeostasis, misfolded or unfolded proteins accumulate within the ER lumen, triggering the unfolded protein response (UPR) [84]. At first, this signaling network serves a protective role. It helps to restore protein balance by stopping overall protein production, increasing the levels of molecular chaperones, and removing misfolded proteins through endoplasmic reticulum-associated degradation (ERAD) [84]. However, when ER stress is sustained or unresolved, the UPR transitions from a pro-survival to a pro-senescent or pro-apoptotic program. This switch is mediated by three primary UPR sensors: protein kinase RNA-like endoplasmic reticulum kinase (PERK), inositol-requiring enzyme 1 (IRE1), and activating transcription factor 6 (ATF6) [85,86,87]. PERK phosphorylates eIF2α, attenuating protein synthesis while selectively enhancing the translation of activating transcription factor 4 (ATF4), which can induce expression of the pro-senescent mediator p21^CIP1^ and the pro-apoptotic factor CHOP (C/EBP homologous protein) [86,87]. Collectively, these UPR arms intersect with canonical senescence pathways, particularly via p53/p21 axis activation, NF-κB signaling, and redox imbalance, promoting a stable growth arrest and contributing to SASP. Chronic ER stress and maladaptive UPR signaling have been increasingly implicated in the pathogenesis of age-related diseases and in the accumulation of senescent cells during tissue aging and degeneration [87].

Aberrant activation of oncogenes such as rat sarcoma viral oncogene homolog (RAS), rapidly accelerated fibrosarcoma (RAF), and mitogen-activated protein kinase kinase(MEK) can paradoxically induce senescence as an early tumor-suppressive mechanism [88]. This oncogene-induced senescence (OIS) is mediated through upregulation of p16 and alternate reading frame (ARF), which inhibit CDK activity and stabilize p53, respectively [89]. While initially protective, prolonged OIS can be detrimental, particularly through the chronic activity of SASP factors, which modulate the tumor microenvironment [90]. Persistent SASP signaling may promote epithelial–mesenchymal transition (EMT), angiogenesis, and inflammation, facilitating tumor progression in later stages [91]. Thus, OIS exemplifies the dual nature of senescence in cancer biology—as both a barrier and an enabler [74].

### 3.2. Gene Expression Dynamics in Cellular Senescence

Cellular senescence is a dynamic and multistep process characterized by progressive changes in gene expression that establish and maintain the senescent phenotype [60]. Transcriptomic analyses, particularly time-series studies of replicative senescence, have revealed distinct temporal phases marked by specific gene expression profiles [92]. In the early stages of senescence, genes linked to growth arrest and metabolic changes are mainly active. In later stages, there is a strong increase in genes that regulate inflammation, immune responses, and remodeling of the extracellular matrix. These changes are key features of SASP [92].

Both replicative senescence and stress-induced premature senescence (SIPS) share common transcriptional pathways, particularly those related to inflammation and immune surveillance [93]. The p53/p21 and p16^INK4a^/Rb signaling axes are central to the initiation and maintenance of growth arrest [94]. However, beyond these canonical pathways, epigenetic remodeling plays a critical role in stabilizing the senescent state [95]. Senescent cells show widespread loss of DNA methylation, along with increased methylation at specific sites. They also display changes in histone modifications that influence how tightly the DNA is packed and how accessible it is for gene expression [96]. For example, loss of the activating histone modification histone 3 lysine 4 trimethylation (H3K4me3) at promoters of cell cycle genes facilitates transcriptional silencing and reinforces proliferative arrest [97].

The tumor suppressor p53 emerges as a key modulator of senescence through its dual role in redox homeostasis and transcriptional regulation [76]. In response to moderate oxidative stress, p53 activates antioxidant defense genes—including *manganese superoxide dismutase (MnSOD)*, *Sestrins*, and *glutathione peroxidase 1*—and metabolic regulators such as *TP53-induced glycolysis and apoptosis regulator (TIGAR)*, *glutaminase 2 (GLS2)*, and *aldehyde dehydrogenase 4 family member A1 (ALDH4A1)*, which promote NADPH production, enhance mitochondrial function, and suppress glycolysis, thereby reducing ROS accumulation [76,98]. Conversely, under severe oxidative damage, p53 shifts its transcriptional program toward promoting apoptosis and senescence by upregulating prooxidant genes (e.g., *NADPH oxidase* and *p53-induced genes 1–13 [PIG1–13]*) and pro-apoptotic mediators such as *Bcl-2-associated X protein (BAX)* and *p53 upregulated modulator of apoptosis (PUMA)*, while repressing antioxidant gene expression [76,98].

Beyond its canonical roles, p53 is intricately linked to the circadian clock, forming a bidirectional regulatory network. p53 can modulate the expression of *Period 2* (PER2), a core component of the circadian machinery, thereby influencing circadian rhythmicity [99]. In turn, circadian clock proteins—such as PER2, BMAL1, and CRY1—can modulate the stability, localization, and activity of p53, affecting its transcriptional responses in cell cycle arrest, DNA repair, and apoptosis [100]. Notably, the circadian regulation of glutaminase 2, one of p53’s target genes, has been observed, suggesting time-of-day-dependent modulation of tumor metabolism and redox balance [100]. In addition, glutathione peroxidase activity has been shown to oscillate in a circadian manner, with peak levels reported around 2 a.m., indicating that enzymatic redox defense is temporally coordinated [101].

Mechanistic target of rapamycin (mTOR) further influences the bifurcation of cell fate downstream of p53 [102]. Maximal activation of p53 inhibits mTOR and favors reversible quiescence, while partial p53 activation permits continued mTOR signaling, pushing cells toward irreversible senescence [102]. These findings highlight the importance of signal intensity and duration in determining cell fate outcomes following stress.

### 3.3. Morphological and Functional Alterations in Senescent Cells

Senescent cells undergo major structural and functional changes that set them apart from cells that are actively dividing or in a resting state [62]. These alterations are not merely phenotypic markers but reflect underlying disruptions in cytoskeletal organization, organelle function, and intracellular signaling [103].

A hallmark of senescence is cellular enlargement and flattening, often accompanied by an irregular, spread morphology [2]. These features result from persistent growth arrest in G1 or G2 phases, during which biosynthetic activity continues, leading to cytoplasmic and nuclear expansion [66]. Enlarged nuclei, multinucleation, and nuclear envelope irregularities, including loss of lamin B1, are frequently observed [97,104,105]. Chromatin undergoes extensive remodeling, characterized by the formation of senescence-associated heterochromatin foci, redistribution of chromosomes, and global epigenetic reprogramming [96,97].

Senescent cells exhibit reorganization of the actin cytoskeleton, with increased formation of stress fibers and focal adhesions mediated by proteins such as ezrin and cofilin-1 [66,106,107,108]. These structural adaptations enhance cell spreading and adherence, possibly contributing to their anti-proliferative barrier function in early tumor suppression.

Several organelles also undergo significant remodeling. Lysosomes are increased in number and volume, reflected by elevated senescence-associated β-galactosidase (SA-β-gal) activity at pH 6.0—a widely used senescence biomarker [109,110]. However, despite their expansion, lysosomal function is impaired, leading to accumulation of nondegradable oxidized lipids, proteins, and lipofuscin [110,111]. Mitochondria in senescent cells are often swollen, fragmented, and dysfunctional, exhibiting increased ROS production, impaired mitophagy, and enhanced but ineffective biogenesis [2,112]. These features contribute to a feed-forward loop that exacerbates oxidative stress and reinforces the senescent state.

Endoplasmic reticulum expansion and stress are also prominent [2,87]. High secretory demand, particularly for SASP factors, strains ER capacity and impairs protein folding, leading to further ROS generation and unfolded protein accumulation [113]. Declines in chaperone expression and disulfide-bond formation capacity reduce proteostasis, contributing to ER dysfunction in aged and senescent cells [114].

Peroxisomes, crucial for ROS detoxification and lipid metabolism, also exhibit structural and functional decline in senescence [3,115]. Their impairment disrupts mitochondrial–peroxisomal crosstalk and worsens redox imbalance, promoting further cellular damage [2]. Restoration of peroxisomal function has been shown to delay senescence onset in experimental models [116].

Functionally, senescent cells are characterized by irreversible cell-cycle arrest, commonly mediated via the p53/p21^Cip1^ and p16^Ink4a^/pRb pathways [60]. While they do not proliferate, senescent cells remain metabolically active and secrete a complex mix of pro-inflammatory, pro-fibrotic, and tissue-remodeling factors known as the senescence-associated secretory phenotype (SASP) [60,117]. These include cytokines (e.g., interleukin-6 [IL-6], interleukin-8 [IL-8]), chemokines (e.g., C-C motif chemokine ligand 2 [CCL2]), growth factors (e.g., platelet-derived growth factor-AA [PDGF-AA], vascular endothelial growth factor [VEGF]), and proteases (e.g., matrix metalloproteinase 1 [MMP1], matrix metalloproteinase 3 [MMP3]) [60].

**Figure 1:**
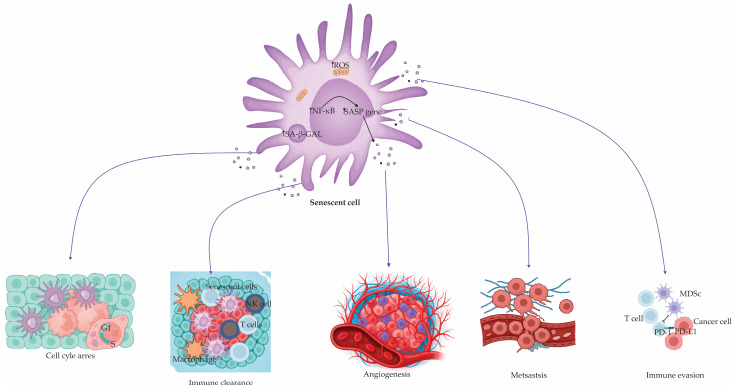
Senescent cell and SASP-mediated effects on tissue and tumor biology. Senescent cells, despite their proliferative arrest, remain metabolically active and secrete an array of bioactive factors collectively termed the senescence-associated secretory phenotype (SASP). SASP factors are transcriptionally regulated predominantly by NF-κB and contribute to a broad spectrum of physiological and pathological outcomes. The figure illustrates how SASP factors influence the following: (1) induction and maintenance of cell cycle arrest; (2) recruitment of immune cells (e.g., NK cells, T cells, macrophages), facilitating immune clearance of tumor cells; (3) promotion of angiogenesis, supporting tumor vascularization; (4) enhancement of metastatic dissemination of tumor cells; (5) immune evasion via mechanisms such as PD-1/PD-L1 axis activation and recruitment of myeloid-derived suppressor cells (MDSCs). This pleiotropic activity underscores the dualistic nature of senescence in health and disease, balancing regenerative processes with the risk of chronic inflammation and malignancy progression.

SASP factors exert pleiotropic effects [118]. They can promote tissue repair, embryonic development, and immune-mediated clearance of senescent or damaged cells [2]. Conversely, chronic SASP expression fosters low-grade systemic inflammation (“inflammaging”), tissue dysfunction, tumor promotion, and metabolic disorders [2]. SASP is regulated via both DDR-dependent mechanisms (via NF-κB and CCAAT/enhancer-binding protein beta [C/EBPβ]) and DDR-independent pathways, such as cyclic GMP-AMP synthase–stimulator of interferon genes (cGAS–STING)-mediated sensing of cytoplasmic DNA fragments derived from compromised nuclear integrity or infection-related stimuli [2,119,120]. An overview of the diverse effects mediated by SASP on tissue homeostasis and tumor progression is illustrated in Figure 1.

Organ-specific senescent phenotypes also vary. For instance, senescent mesenchymal stem cells (MSCs) adopt a flattened, polygonal morphology, while senescent microglia become hypertrophic with shortened processes [121]. In aging kidneys, tubular epithelial cells display characteristic senescent markers alongside decreased proliferative capacity [122]. These morphological and functional features reflect the localized responses to stress and damage in different tissues. A summary of the molecular triggers, cellular changes, and clinical implications of senescence is provided in Table 2.

## 4. Oxidative Stress as a Central Driver and Sustainer of Cellular Senescence

Oxidative stress represents a pivotal biological force that not only initiates but also perpetuates cellular senescence [123]. The excessive accumulation of ROS disrupts redox homeostasis and induces macromolecular damage, particularly within DNA and mitochondria, thereby activating stress-responsive signaling pathways that enforce cell cycle arrest and senescence [31,124]. Among the earliest and most critical consequences of oxidative stress is the activation of DDR, often via ROS-induced lesions at telomeric sites [125,126,127]. This leads to the stabilization of p53 and the transcriptional upregulation of cyclin-dependent kinase inhibitors such as p21^CIP1^ and p16^INK4a^, culminating in durable growth arrest and the formation of SAHF [128,129,130].

Mitochondrial dysfunction is a critical driver and hallmark of cellular senescence [131]. Damage to mitochondrial DNA (mtDNA), which lacks protective histones and has limited repair capacity, compromises the integrity of respiratory chain complexes and impairs oxidative phosphorylation. This dysfunction results in inefficient electron transfer and increased electron leakage, particularly at complexes I and III, leading to the excessive generation of ROS [132,133]. The accumulation of ROS further damages mitochondrial and nuclear components, establishing a self-perpetuating loop of oxidative stress that stabilizes the senescent phenotype [132,133].

Alongside mitochondrial dysfunction, impaired peroxisomal function exacerbates redox imbalance. Peroxisomes play a vital role in detoxifying ROS, especially hydrogen peroxide, through catalase activity. Their insufficiency in senescent cells contributes to sustained oxidative damage and the reinforcement of redox-sensitive signaling pathways involved in growth arrest and SASP induction [134].

In response to mitochondrial distress, senescent cells activate compensatory pathways aimed at restoring energy homeostasis. One such response involves mitochondrial biogenesis driven by activation of the ataxia-telangiectasia mutated (ATM) kinase, which, through downstream effectors including protein kinase B (Akt) and mechanistic target of rapamycin complex 1 (mTORC1), promotes transcriptional coactivators such as Peroxisome proliferator-activated receptor gamma coactivator 1-alpha (PGC-1α) [135,136]. This axis attempts to increase mitochondrial mass and function, but in the context of senescence, the biogenesis process is often dysfunctional or insufficient. Concurrently, a metabolic shift toward aerobic glycolysis (Warburg-like effect) occurs, favoring ATP production independent of the impaired electron transport chain [135,136].

Despite these adaptive responses, senescent cells remain in a state of chronic energetic stress, characterized by altered NAD^+^/NADH ratios, sustained AMPK activation, and elevated ROS levels. Namely, ROS also activate metabolic checkpoints such as AMPK, which senses altered AMP/ATP and NAD^+^/NADH ratios [137]. AMPK engagement contributes to senescence by stabilizing p21 and p16 mRNA transcripts through inhibition of the RNA-binding protein human antigen R (HuR) [138,139]. This stabilization promotes the activation of p53 and Rb pathways, reinforcing the senescent arrest program [139].

Importantly, oxidative stress-induced senescence is not a passive consequence but a tightly regulated process supported by self-sustaining signaling loops [111]. One example of this feedback loop includes several key molecules: cyclin-dependent kinase inhibitor 1A (CDKN1A or p21), growth arrest and DNA damage-inducible alpha (GADD45A), mitogen-activated protein kinase 14 (MAPK14), growth factor receptor-bound protein 2 (GRB2), and transforming growth factor-beta (TGF-β). In this pathway, ROS help to sustain DDR signaling and promote continued expression of p21, reinforcing the senescent state [140,141]. This leads to a stable senescent state that is difficult to reverse [140]. Moreover, ROS actively participate in shaping SASP, a pro-inflammatory secretome that exacerbates tissue dysfunction, propagates paracrine senescence, and promotes chronic inflammation [3].

Compelling experimental data further reveal the reciprocal crosstalk between mitochondrial and telomeric ROS [142]. ROS localized to mitochondria can induce telomere shortening and genomic instability, while telomere-specific oxidative damage reciprocally impairs mitochondrial function, compounding cellular stress [143,144]. This bidirectional signaling underlines the systemic nature of redox imbalance in senescence.

In sum, oxidative stress is not merely a correlate but a causative and sustaining force in cellular senescence. Through its roles in DNA damage, mitochondrial impairment, and pro-senescent signaling, ROS are deeply embedded in the molecular circuitry of aging and age-related disease. Therapeutic strategies aimed at restoring redox homeostasis hold promise for mitigating senescence and its pathological consequences.

### 4.1. DNA Damage Response, Oxidative Stress, and the Reinforcement of Senescence

The DNA damage response (DDR) is a fundamental signaling cascade that not only initiates but also sustains cellular senescence, acting at the intersection of nuclear genome integrity, oxidative stress, and mitochondrial function [140,145]. In response to persistent genotoxic stress, such as double-strand DNA breaks induced by ROS, radiation, or replication stress, DDR is activated through key sensors like ATM and ATR, leading to the phosphorylation of downstream effectors such as CHK1, CHK2, and p53 [130]. This cascade triggers the transcriptional upregulation of cell cycle inhibitors, including p21^CIP1^ and p16^INK4a^, establishing growth arrest [130].

However, DDR signaling extends beyond nuclear checkpoint control. Sustained DDR activity promotes mitochondrial remodeling and functional decline, leading to increased production of ROS [140]. Mechanistically, DDR activation engages a signaling axis involving growth arrest and DNA damage-inducible protein 45 (GADD45), which activates p38 mitogen-activated protein kinase (p38 MAPK). This, in turn, signals through GRB2 and TGF-β pathways, resulting in transcriptional and metabolic reprogramming that enhances mitochondrial ROS production [140].

The elevated ROS levels not only damage mitochondrial and cytoplasmic components but also further compromise nuclear DNA integrity [7]. This establishes a self-amplifying feedback loop in which ROS perpetuate DNA lesions, thereby sustaining DDR signaling and maintaining DNA damage foci, including 53BP1- and γH2AX-marked chromatin domains. These persistent DDR foci function as epigenetic scaffolds that stabilize the senescent phenotype by continuously enforcing checkpoint signaling and facilitating the secretion of pro-inflammatory SASP components [140].

Importantly, pharmacological inhibition of ROS leads to restoration of mitochondrial membrane potential, while combined suppression of ROS and mTOR signaling can further mitigate senescence-associated mitochondrial defects and DNA double-strand breaks [146,147,148]. These findings underscore the role of mitochondrial ROS as active contributors to DDR persistence and senescence maintenance.

The downstream signaling cascade of DDR—including ATM activation and subsequent Akt/mTORC1 phosphorylation—serves not only to regulate DNA repair but also to promote mitochondrial biogenesis via peroxisome proliferator-activated receptor gamma coactivator 1-beta (PGC-1β) [149]. This adaptive response, though initially compensatory, paradoxically supports the senescent phenotype by reinforcing mitochondrial-derived ROS production and metabolic burden [149].

### 4.2. Oxidative Stress and Its Modulatory Role in Senescence-Associated Signaling Pathways

Oxidative stress plays a crucial role in orchestrating the molecular signaling that underpins cellular senescence [150]. One of the hallmark features of senescent cells is irreversible cell cycle arrest, which is accompanied by a broad transcriptional suppression of genes involved in mitotic progression, DNA replication, and chromosomal segregation [60]. Reactive oxygen species, generated under oxidative stress, profoundly affect the expression and activity of these gene networks, thereby reinforcing the senescence phenotype [151].

**Figure 2:**
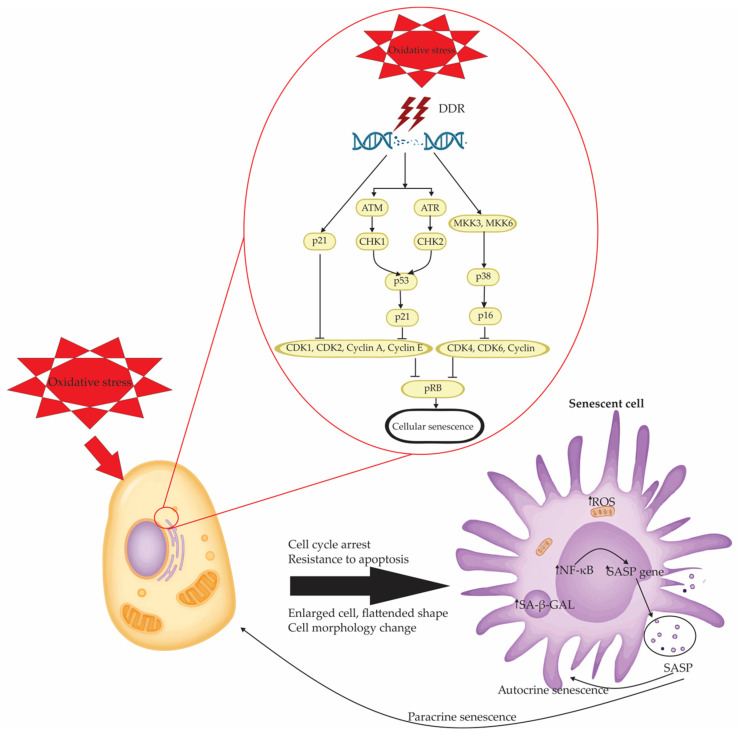
Mechanistic pathways linking oxidative stress to cellular senescence and SASP activation. Oxidative stress induces cellular senescence primarily through DNA damage response (DDR) activation, leading to a cascade of molecular events that enforce cell cycle arrest. The central DDR machinery involves the ATM/checkpoint kinase 2 (CHK2)/p53/p21 axis and the Rad3-related (ATR)/checkpoint kinase 1 (CHK1)/mitogen-activated protein kinase kinase 3 (MKK3)–p38/p16 pathway. These converging routes inhibit cyclin-dependent kinases (CDK1, CDK2, CDK4, CDK6) and cyclins (A, E, D), ultimately suppressing phosphorylation of the retinoblastoma protein (pRB) and halting cell cycle progression. This checkpoint blockade ensures a stable senescent phenotype characterized by resistance to apoptosis, cellular enlargement, morphological changes, and flattened cell shape. Additionally, senescent cells exhibit increased senescence-associated β-galactosidase (SA-β-GAL) activity and heightened mitochondrial ROS (↑ROS) production, which further amplifies redox imbalance. NF-κB activation drives the transcription of SASP genes, culminating in the secretion of diverse inflammatory and tissue-remodeling factors. These SASP components perpetuate senescence in an autocrine manner and propagate paracrine senescence in surrounding cells, thereby contributing to tissue dysfunction, chronic inflammation, and tumorigenic processes.

Oxidative stress is a strong trigger of cellular senescence. It mainly acts by causing lasting DNA damage and disturbing the balance of the cellular redox system. Among the principal mediators of this response are the p53–p21 and p16^INK4a^–Rb tumor suppressor pathways, which function as key transcriptional and cell cycle checkpoints sensitive to redox perturbations [152,153]. Oxidative ROS trigger DNA lesions that activate DDR. This cascade begins with the activation of ATM or ATM and ATR kinases, which phosphorylate checkpoint kinases CHK2 and CHK1, respectively [154]. These kinases, in turn, stabilize p53 and promote its transcriptional activity, including the upregulation of p21^CIP1^, a CDK inhibitor that disrupts CDK2 and CDK1 activity, thereby blocking G1/S and G2/M cell cycle transitions [155]. Simultaneously, ROS can upregulate p16^INK4a^, either directly via redox-sensitive transcriptional programs or indirectly through p38 MAPK activation downstream of DDR. p16^INK4a^ inhibits CDK4 and CDK6, preventing the phosphorylation of pRb and maintaining Rb in its active, hypophosphorylated state. This results in the transcriptional silencing of E2F target genes, which are required for S-phase entry, thus enforcing durable growth arrest [151]. Together, these pathways suppress the activity of multiple cyclin–CDK complexes, including those involving cyclins D, E, and A, thereby locking the cell in a senescent state [151]. The interplay between oxidative stress, DNA damage response, and the p53–p21 and p16–Rb pathways in driving cellular senescence is schematically represented in Figure 2.

A notable redox-sensitive effector in this context is thrombospondin-1 (TSP1), a matricellular glycoprotein that exerts multiple effects on cellular homeostasis and is upregulated in senescent cells [156]. TSP1 expression is triggered by DNA damage, inflammatory cytokines like TGF β1, and increased oxidative stress, which places it downstream of important signals that initiate senescence such that itacts as a feedback amplifier [157,158,159]. Once secreted, TSP1 binds to its receptor CD47 (also known as integrin-associated protein), triggering the activation of NADPH oxidase 1 (Nox1) [156]. This leads to increased ROS production, which amplifies nuclear p53 signaling, upregulates p21^CIP1/WAF1^, and enforces cell cycle arrest. This TSP1–CD47–Nox1 axis thereby strengthens the DNA damage response and sustains the senescence phenotype.

Importantly, this axis also engages in crosstalk with other redox-regulatory systems. For example, TSP1-induced CD47 activation inhibits nitric oxide signaling, a crucial antioxidant and vasoprotective pathway, further shifting the redox balance in favor of oxidative stress [160]. This contributes not only to intracellular DNA damage and senescence propagation but also to extracellular microenvironment remodeling, including impaired angiogenesis and vascular dysfunction, particularly relevant in aging tissues [160]. Overall, TSP1 acts as both a downstream effector and an upstream amplifier of oxidative stress and p53-driven senescence, forming a feed-forward loop that reinforces redox imbalance and permanent growth arrest.

Furthermore, exposure to environmental stressors, such as polystyrene nanoplastics (PS-NPs), has been shown to provoke oxidative stress via NADPH oxidase 2 (Nox2) activation [161]. This culminates in enhanced activation of both the p53–p21 and Rb–p16 pathways, contributing to premature senescence in spermatogenic cells [162].

At the transcriptional level, oxidative stress alters the expression of genes involved in vesicular trafficking, cell adhesion, and membrane receptor signaling, reflecting the adaptive remodeling of senescent cells [151]. Additionally, senescent cells often exhibit increased expression of anti-apoptotic B-cell lymphoma 2 (BCL-2) family proteins (e.g., BCL-2, B-cell lymphoma-extra large [BCL-XL], BCL-w), while pro-apoptotic mediators such as caspase-3 are downregulated, supporting their long-term survival despite accumulating damage [151].

### 4.3. Mitochondrial Damage and Oxidative Stress in Senescence Induction

Mitochondria are central hubs in regulating redox homeostasis, and their dysfunction is intimately linked to the onset and progression of cellular senescence [163]. One of the key mechanisms involves damage to mtDNA, which is highly susceptible to oxidative insults due to its close proximity to the electron transport chain and limited repair capacity [163]. Elevated reactive oxygen species, either intrinsically generated or induced by external stressors, can directly damage mtDNA or exacerbate mitochondrial dysfunction via alterations in telomerase reverse transcriptase (TERT) activity and modulation of tumor suppressor pathways such as p53 and Ras [164].

Persistent mitochondrial dysfunction is a hallmark of senescent cells and plays a central role in reinforcing the senescence program. Damaged mitochondria constantly produce high levels of ROS. This creates a self-sustaining cycle where oxidative stress further harms the mitochondria, leading to ongoing DNA damage and activation of redox-sensitive signaling pathways [163]. This sustained ROS production not only drives initial senescence induction but also actively contributes to the maintenance of the senescent phenotype.

One critical mechanism by which ROS mediate long-term growth arrest involves the activation of the GADD45–p38 mitogen-activated protein kinase (p38 MAPK)–growth factor receptor-bound protein 2 (GRB2)–transforming growth factor beta receptor 2 (TGFBR2)–TGF-β axis [140]. The GADD45 family, activated downstream of persistent DDR and oxidative stress, promotes phosphorylation of p38 MAPK, a key regulator of stress-responsive gene expression [165]. Activated p38 MAPK interacts with GRB2 and enhances TGF-β signaling through increased expression and activation of TGFBR2 [166].

TGF-β signaling, in this context, exerts strong anti-proliferative effects by inducing cyclin-dependent kinase inhibitors (such as p15^INK4b^ and p21^CIP1^) and promoting chromatin remodeling at E2F target gene promoters, thereby stabilizing Rb-mediated cell cycle arrest [166]. Importantly, this signaling cascade is not merely a transient checkpoint but constitutes a positive-feedback mechanism that ensures the irreversibility of senescence once established. The convergence of ROS production, DDR persistence, and TGF-β–mediated signaling creates a robust network that sustains the senescent state, rendering it resistant to mitogenic cues and apoptosis [3,140].

Thus, mitochondrial damage and the associated oxidative stress are not merely byproducts of cellular aging but active participants in the molecular orchestration of senescence, with implications for aging-related pathologies and therapeutic targeting.

### 4.4. Crosstalk Between Oxidative Stress, SASP, and the Nrf2–mTOR Axis

Oxidative stress is a key upstream signal that modulates both the onset and persistence of SASP [2,90]. Reactive oxygen species not only initiate DNA damage responses but also amplify SASP gene expression by activating redox-sensitive transcription factors such as NF-κB and C/EBPβ [2,167,168]. These transcriptional regulators drive the production of proinflammatory mediators like TNF-α, IL-1β, and IFN-γ, which can further propagate oxidative stress in neighboring cells, creating a self-reinforcing loop that sustains tissue-level senescence and inflammation [169,170].

Within this oxidative context, the Nrf2 transcription factor plays a dual role [171]. Under oxidative pressure, Nrf2 translocates into the nucleus and induces a specific arm of SASP, designated the Nrf2-induced secretory phenotype (NISP) [172]. This transcriptional program selectively promotes immune surveillance by recruiting CCR2-positive monocytes, facilitating the clearance of damaged or senescent cells [173]. Thus, Nrf2 acts as a protective tumor suppressor by curbing the long-term persistence of potentially harmful senescent cells [174].

Recent studies support the notion that Nrf2 directly and indirectly regulates mTOR activity, positioning the Nrf2–mTOR axis as a central redox–senescence regulatory hub [175]. At the transcriptional level, in vitro and organismal models have shown that Nrf2 activation can drive mTOR gene expression by binding to its promoter region, thereby promoting mTORC1 signaling even under basal conditions [176]. Nrf2 also enhances mTOR signaling through upregulation of RagD, a small GTPase essential for lysosomal targeting and activation of mTORC1 in nutrient-sensing pathways [177]. These mechanisms suggest that the oxidative stress-responsive Nrf2 participates in metabolic and growth regulatory feedback loops by tuning mTOR activity via transcriptional and post-translational modulators.

Conversely, mTOR stabilizes Nrf2 by inhibiting its degradation through suppression of the β-TrCP E3 ubiquitin ligase pathway [177]. Under normal conditions, β-TrCP recognizes phosphorylated Neh6 domain motifs on Nrf2 (e.g., via GSK-3β), leading to Cullin-1-mediated Nrf2 degradation independent of Keap1 [178]. mTOR activation suppresses β-TrCP expression/activity, thereby enhancing Nrf2 nuclear accumulation and antioxidant gene transcription, as demonstrated in pathological models (e.g., diabetic nephropathy) [17,179,180,181].

Finally, the feedback architecture of this axis is particularly relevant in aging and senescence. Persistent mTORC1 hyperactivity, common in aging tissues, can maintain SASP, while concurrently reinforcing Nrf2 stabilization [78]. Depending on the cellular context, Nrf2 may drive either pro-survival antioxidant responses or immune-mediated clearance via SASP regulation [182]. Together, this reciprocal regulation integrates redox stress sensing (via Nrf2) with metabolic and growth signaling (via mTOR), reinforcing senescence through feed-forward loops and offering a coherent mechanistic framework for targeting this axis in age-related disease and cancer.

### 4.5. MicroRNAs as Modulators and Biomarkers of Oxidative Stress-Driven Senescence

MicroRNAs (miRNAs) have emerged as crucial post-transcriptional regulators of gene expression that participate in both the onset and maintenance of cellular senescence, particularly in the context of oxidative stress [183,184,185]. A variety of miRNAs, including miR-21, miR-22, miR-34a, miR-146a, and members of the miR-17-92 and miR-200 families, are differentially expressed in senescent cells and aged tissues [185]. Importantly, oxidative stress can both influence miRNA biogenesis and modulate the expression profiles of specific miRNAs, thereby contributing to senescence induction [185]. Cellular oxidative stress can impair the miRNA biogenesis machinery, notably through ROS-mediated inactivation of protein tyrosine phosphatase 1B (PTP1B), which leads to phosphorylation and functional inhibition of Argonaute 2 (AGO2). This disruption compromises miRNA loading into the RNA-induced silencing complex (RISC) and represents a critical upstream regulatory checkpoint in the initiation of senescence, as demonstrated in RAS-induced premature senescence models. In this context, ROS serve as both inducers of DNA damage and as upstream modulators of miRNA silencing efficiency, coupling oxidative stress directly to the failure of post-transcriptional regulation that enforces cell cycle arrest and senescence propagation [186].

At the same time, specific miRNAs act as downstream amplifiers and fine-tuners of oxidative stress-induced senescence programs. Reactive oxygen species directly upregulate miR 210 and miR 494, which impair mitochondrial function, inhibit autophagy, and exacerbate DNA damage via downregulation of key repair proteins such as the MRN complex, thereby reinforcing senescence hallmarks [3,187].

Additionally, families of miRNAs—including miR-21, miR-34a, miR-146a, and members of the miR-17-92 and miR-200 clusters—are transcriptionally altered in response to oxidative stress and contribute to feedback loops that regulate redox homeostasis, inflammatory signaling, and cell cycle exit in aging cells and tissues [188]. Beyond their intracellular regulatory roles, miRNAs are secreted into the circulation, where they are stabilized within extracellular vesicles or bound to carrier proteins, enabling them to act as intercellular signaling molecules and accessible biomarkers [189]. Numerous studies have demonstrated that circulating miRNAs—such as miR-126, miR-93, miR-21, and miR-146a—are altered in aging and age-related diseases [190,191]. Notably, miR-21, which is elevated in age-associated pathologies and various cancers, is found at lower levels in centenarians, indicating a possible role in healthy aging and longevity prediction [192].

A comprehensive overview of oxidative stress-driven mechanisms of senescence and their clinical implications is presented in Table 3.

## 5. Senotherapy: Targeting Senescent Cells for Healthspan Extension

Senotherapy encompasses a group of therapeutic strategies specifically designed to counteract the deleterious effects of cellular senescence by either eliminating senescent cells (senolytics) or modulating their secretory profile (senomorphics) [193]. As senescent cells accumulate in tissues with age or in response to stress, they exert adverse effects primarily through the secretion of pro-inflammatory and matrix-degrading factors collectively known as SASP [118]. Persistent SASP signaling not only promotes chronic inflammation but also drives the senescence of neighboring cells, thereby amplifying tissue dysfunction in a paracrine manner [2].

Senescent cells have been implicated in the pathogenesis of a wide spectrum of age-associated disorders, including neurodegenerative diseases, cardiovascular dysfunction, diabetes, osteoporosis, and chronic renal and hepatic diseases [194]. Their accumulation at sites of pathology is increasingly recognized as a contributor to disease progression and organ failure [195]. Consequently, the targeted elimination of these cells has emerged as a promising strategy to restore tissue homeostasis and delay age-related decline [195].

Given the role of oxidative stress in reinforcing the senescent state and driving SASP production, future senotherapy efforts may benefit from combined regimens that also modulate redox balance, offering synergistic benefits in controlling the detrimental consequences of cellular senescence.

### 5.1. Senolytic Strategies: Selective Clearance of Senescent Cells to Restore Tissue Integrity

The therapeutic targeting of senescent cells (SNCs) through senolytics has emerged as a promising strategy to counteract chronic inflammation, tissue dysfunction, and age-associated diseases [196]. Unlike conventional therapies, senolytics act by selectively inducing apoptosis in senescent cells that evade immune clearance through activation of pro-survival pathways [196,197]. These interventions aim not only to reduce the burden of dysfunctional cells but also to mitigate the proinflammatory milieu driven by SASP [193].

Initial breakthroughs came with the identification of dasatinib and quercetin (D+Q) as effective senolytic agents [198]. This combination targets tyrosine kinases and phosphoinositide 3-kinase (PI3K)-dependent survival signaling and has shown efficacy in both preclinical models and early-phase clinical trials for conditions such as idiopathic pulmonary fibrosis and cancer [199,200,201,202]. Intermittent dosing of D+Q has been shown to improve physical function and reduce senescence markers in treated patients [193,200]. Moreover, in ovarian cancer models, D+Q mitigated chemotherapy-induced senescence in adipose stromal cells, thereby inhibiting metastatic dissemination [201].

The BCL-2 protein family represents a key target in senescence due to their upregulation in SNCs [203]. Navitoclax (ABT-263), a potent inhibitor of BCL-2, BCL-XL, and BCL-W, promotes apoptosis in senescent cells, particularly in chemotherapy-induced models of resistant cancers such as pancreatic and breast tumors [200,204,205]. However, its clinical translation is limited by hematologic toxicity, particularly thrombocytopenia. Similar limitations apply to its structural analog, ABT-737 [206]. To circumvent these drawbacks, ongoing research is exploring tissue-specific delivery vectors, intermittent regimens, and novel inhibitors with enhanced selectivity.

Flavonoids such as quercetin, fisetin, apigenin, and resveratrol act as senolytic compounds by interfering with ROS signaling, SASP induction, and the cell cycle arrest pathway. Quercetin reduces ROS and inflammatory cytokines, upregulates antioxidant responses via Nrf2, and downregulates markers of senescence such as p16 and p53 while promoting SOD and CAT expression; it also modulates microRNA-155–SIRT1–NF-κB signaling to inhibit SASP-driven inflammation [207,208,209,210]. Fisetin similarly diminishes oxidative stress, promotes senolytic clearance, mimics caloric restriction, and extends lifespan in vivo—though its effects depend on timing relative to oxidative challenge [208,211,212].

Apigenin, a flavone, directly suppresses SASP through inhibition of IL-1α–IRAK1/4, p38-MAPK, and NF-κB signaling in senescent fibroblasts, reducing secretion of pro-inflammatory cytokines without necessarily inducing senescent-cell death [210,213]. Other compounds under evaluation include cardiac glycosides (digoxin, ouabain), glutaminase inhibitors, and mTOR inhibitors like rapamycin, which indirectly suppress SASP signaling via modulation of NF-κB translation [214,215,216].

Mechanistically distinct approaches, such as the forkhead box O4–D-retro-inverso (FOXO4-DRI) peptide, disrupt the FOXO4–p53 interaction, freeing p53 to trigger senescence-specific apoptosis [62,217]. Likewise, heat shock protein 90 (HSP90) inhibitors destabilize chaperone networks that sustain senescent cell viability [218].

Despite these advances, several challenges hinder clinical translation. Senescent cells are heterogeneous across tissues and disease contexts, displaying variable dependency on pro-survival pathways [219]. As such, a senolytic agent effective in one setting may be ineffective—or deleterious—in another. Furthermore, repeated or widespread senescent cell clearance may impair regenerative capacity or provoke compensatory cell damage [2]. Kowald and colleagues have emphasized that while senolytics remove harmful cells, their inappropriate use may accelerate tissue dysfunction [220].

### 5.2. Senomorphics: Modulating the Harmful Phenotype of Senescent Cells

Senomorphics represent an alternative and increasingly valued strategy in the therapeutic modulation of cellular senescence [221]. Rather than eliminating senescent cells, senomorphic agents aim to suppress or reshape the senescence-associated secretory phenotype, a key driver of chronic inflammation and tissue degeneration in aging [193]. By preserving the structural integrity of SNCs while mitigating their deleterious paracrine signaling, senomorphics offer a refined approach for managing age-related pathologies, especially in tissues where transient senescence plays protective or regenerative roles [90,193,222].

SASP consists of a dynamic and complex mix of pro-inflammatory cytokines (e.g., IL-6, IL-8, IL-1β), chemokines, matrix metalloproteinases, growth factors, and bioactive lipids [90]. It is governed by interconnected signaling axes, including NF-κB, mTOR, Janus kinase/signal transducer and activator of transcription (JAK/STAT), and p38 MAPK [223,224]. Persistent SASP activity promotes local inflammation, stem cell dysfunction, and propagation of senescence to surrounding cells, thereby contributing to conditions such as cancer, osteoarthritis, and neurodegeneration [90,169]. Importantly, the composition and function of SASP evolve temporally: early SASP components can mediate tissue repair and immune evasion, while chronic SASP tends to drive inflammatory deterioration [90].

A variety of agents have been shown to suppress SASP activity. Rapamycin and its analogs (rapalogs), which inhibit mTOR signaling, reduce SASP output by blocking IL-1α translation, a key SASP initiator [193]. Similarly, JAK inhibitors such as ruxolitinib and baricitinib effectively blunt IL-6/IL-8-driven SASP propagation and restore tissue homeostasis in both preclinical and clinical settings [225,226]. Metformin, beyond its antidiabetic action, activates AMPK and downregulates mTOR, thereby exerting senomorphic effects and reducing low-grade inflammation linked to aging and metabolic disease [227]. Other phytochemicals, including apigenin, kaempferol, and rutin, suppress SASP expression via interference with ATM–TRAF6–HIF1α signaling or inhibition of MAPK-dependent transcription [228,229,230].

In addition to synthetic and plant-derived compounds, newer targets for senomorphic intervention are emerging [231]. Inhibitors of nicotinamide phosphoribosyltransferase (NAMPT), such as FK866, interfere with nicotinamide adenine dinucleotide (NAD^+^) biosynthesis and suppress therapy-induced senescent cancer stem-like cells [232]. Certain compounds can reduce SASP without causing cell death. These include inhibitors of p38 MAPK, bromodomain and extraterminal domain (BET) proteins, and O-linked N-acetylglucosamine glycosylation (O-GlcNAcylation) [233,234]. Although some glucocorticoids show senomorphic activity via NF-κB inhibition, their systemic immunosuppressive effects limit long-term utility [193].

Importantly, the context-dependent roles of SASP require a nuanced therapeutic approach. While suppressing chronic SASP is desirable, indiscriminate inhibition may interfere with its beneficial roles in wound healing and tumor surveillance [235]. Thus, temporally and spatially regulated senomorphic therapies are under development, often guided by tissue-specific delivery systems and AI-driven compound optimization [193]. Precision in targeting SASP regulators is essential to selectively suppress deleterious factors while preserving reparative or immune-supportive elements [193].

In clinical translation, senomorphics may be used alone or in combination with senolytics [193]. Sequential regimens—such as SASP suppression prior to senescent cell clearance, or post-clearance suppression to prevent SASP propagation—are under active investigation [193,236]. Moreover, the absence of non-invasive biomarkers to assess the SASP burden in vivo remains a major challenge for stratifying patients and tracking therapeutic response.

### 5.3. Redox Modulation in Senescence Management

Oxidative stress plays a central role in the initiation and propagation of cellular senescence [111]. The duality of reactive oxygen species—as both signaling molecules and sources of cellular damage—has positioned redox modulation as a key therapeutic avenue in senescence-targeted interventions [12]. Depending on the context, redox-based therapies can either amplify ROS to induce senescence or cell death in tumor cells, or attenuate ROS to prevent stress-induced senescence and chronic inflammation in aging tissues [237,238].

One compound of interest is resveratrol, a polyphenolic antioxidant found in grapes and berries, which exhibits dose-dependent dual activity [239]. At low concentrations, it suppresses inflammation-induced senescence, particularly in nucleus pulposus cells, while at higher doses, it paradoxically induces oxidative stress and DNA damage, promoting senescence or apoptosis in cancer cells via mitochondrial disruption and stress-activated kinases [239,240,241]. Clinical findings further support the redox-dependent actions of resveratrol; in combination with copper, it acts as a prooxidant that reduces chemotherapy-associated toxicity in gastric cancer patients [242].

Ginsenoside Rh2, derived from ginseng, has been shown to modulate redox signaling by attenuating NF-κB/IL-8-driven SASP in doxorubicin-resistant breast cancer models, thereby inhibiting cell proliferation and restoring chemosensitivity [243]. Similarly, apigenin, a dietary flavonoid, exerts its senescence-inducing effects via sustained oxidative signaling through p21 activation, independent of canonical p53 or p16-Rb pathways, suggesting a promising role in redox-based sensitization of colorectal cancer cells to therapy [244].

A wide spectrum of natural compounds—such as phloretin, genistein, sulforaphane, quercetin, silybin, and curcumin analogs—have demonstrated potential to modulate oxidative stress and induce senescence selectively in cancerous cells [244]. Several of these agents have advanced into preclinical validation, highlighting their promise as redox-active senotherapeutics with both antioxidant and prooxidant capacities [244].

In parallel, antioxidant strategies are being explored to mitigate the detrimental effects of senescence in non-cancerous tissues [245,246]. Enzymatic mimetics of superoxide dismutase and glutathione peroxidase, as well as Nrf2 activators like sulforaphane, have shown efficacy in restoring redox homeostasis and limiting oxidative DNA damage [247,248,249]. Inhibitors of NADPH oxidase (e.g., diphenyleneiodonium) directly reduce ROS production, dampening SASP and its associated inflammatory cascade [250].

Collectively, redox modulation represents a versatile platform in senotherapy—whether by enhancing ROS to selectively eliminate damaged or malignant cells or by curbing oxidative stress to preserve tissue integrity and suppress chronic inflammation. The therapeutic outcome hinges on precise control of ROS levels, cell context, and the integration of redox pathways with key senescence effectors such as p21, p53, and NF-κB.

### 5.4. Gene Therapy Approaches in Senotherapeutics

Gene therapy has recently gained momentum as a precision strategy to combat cellular senescence by directly targeting the genetic regulators of aging and stress responses [251]. This approach harnesses advanced molecular tools to either restore function in cells undergoing premature senescence or eliminate senescent cells that contribute to tissue dysfunction and chronic disease [251].

Interventions aimed at modulating the expression of key cell cycle regulators, such as CDK2 and the retinoblastoma (RB) tumor suppressor, have demonstrated the potential to delay senescence onset and maintain proliferative competence in aging cells [252]. By correcting or compensating for disruptions in these pathways, gene therapy may preserve genomic integrity and cellular plasticity, particularly in high-turnover tissues susceptible to degenerative changes [253].

An alternative application, known as suicide gene therapy, is being explored to selectively ablate senescent cells through the activation of endogenous apoptotic mechanisms [62]. In this context, senescence-specific promoters drive the expression of cytotoxic genes only in cells exhibiting a senescent phenotype, thus enabling their precise removal while sparing surrounding healthy tissue. This targeted approach offers a means to reduce senescent cell burden without the systemic effects associated with conventional senolytic drugs [62].

By integrating gene repair and targeted ablation strategies, gene therapy provides a versatile and highly customizable platform for senescence management. Its therapeutic potential is particularly relevant for age-related pathologies, such as cataracts, neurodegeneration, and fibrotic disorders, where the accumulation of senescent cells disrupts tissue architecture and function. As delivery systems and vector safety continue to improve, gene therapy is poised to become a cornerstone of next-generation senotherapeutics aimed at extending healthspan and delaying the molecular hallmarks of aging.

### 5.5. Harnessing the Immune System for Senescent Cell Clearance

Recent advancements have revealed the immune system’s essential role in identifying and eliminating senescent cells, opening the door to immunotherapeutic strategies aimed at promoting healthy aging and enhancing cancer treatment [253]. Senescent cells often display altered expression of surface proteins, making them susceptible to immune surveillance mechanisms that can be therapeutically exploited [254].

One promising approach involves engineering chimeric antigen receptor (CAR) T cells to specifically recognize and destroy senescent cells expressing surface molecules such as urokinase-type plasminogen activator receptor (uPAR) [255]. Preclinical models have demonstrated that uPAR-targeted CAR-T cells efficiently eliminate senescent tumor cells induced by MEK and CDK4/6 inhibition, thereby suppressing tumor progression in lung cancer models [255].

Natural killer (NK) cells also participate in senescent cell clearance through recognition of upregulated ligands for activating receptors like natural killer group 2 member D (NKG2D) [256]. Enhancing NKG2D-mediated signaling may potentiate NK cell cytotoxicity against senescent targets and contribute to improved immune surveillance in aging tissues and malignancies [256,257].

Checkpoint blockade therapies, widely used in oncology, are now being considered in the context of senescence [258]. Senescent cells often overexpress programmed death-ligand 1 (PD-L1), contributing to immune evasion and chronic inflammation [259]. Thus, immune checkpoint inhibitors targeting PD-L1 could selectively restore immune-mediated clearance of senescent cells, particularly in aged or tumor-bearing hosts [260,261].

Furthermore, manipulation of the tumor microenvironment to improve immunogenicity of senescent cells is a developing area of interest. Epigenetic repression of pro-inflammatory SASP genes—such as those encoding CCL2 and CXCL9/10—by enhancer of zeste homolog 2 (EZH2) has been shown to dampen immune cell infiltration in pancreatic cancer models [262]. Pharmacologic inhibition of EZH2 reactivates SASP gene expression, enhances chemokine-mediated recruitment of NK and T cells, and facilitates the immune-mediated elimination of therapy-induced senescent (TIS) cells [262].

Collectively, these findings support the development of immune-based senotherapeutics that selectively augment the immune system’s ability to target senescent cells. Such strategies hold promise not only for cancer therapy but also for mitigating the deleterious effects of senescent cell accumulation in age-related diseases. A comprehensive overview of senotherapeutic strategies, their mechanisms, and clinical implications is summarized in Table 4.

## 6. Conclusions

Cellular senescence stands at the crossroads of oxidative stress, mitochondrial dysfunction, and chronic inflammation. Excessive ROS damage organelles and DNA, triggering a persistent DNA damage response (DDR) and reinforcing SASP, which can shift senescence from a protective arrest to a driver of tissue degeneration. This transition is regulated by the nuclear factor Nrf2 and mechanistic target of rapamycin (mTOR) axis, which integrates metabolic signals with antioxidant defenses to determine whether senescence remains beneficial or becomesharmful through a process known as inflammaging. Current research is shifting from general antioxidant therapies to precision senotherapeutics. These include redox modulators combined with senolytics or senomorphics, senescence-targeted gene editing, promoter-driven suicide constructs, and engineered CAR-T or NK cells targeting uPAR or NKG2D. The goal is to eliminate or reprogram senescent cells while preserving tissue regeneration. However, three key challenges remain: addressing the variability of senescent cells across tissues and diseases, managing the dual roles of SASP, and developing non-invasive, ROS-sensitive biomarkers—such as circulating microRNA panels or imaging agents—to guide treatment selection, monitor response, and adjust dosing.

## 7. Future Directions

Despite major strides in elucidating the molecular underpinnings of oxidative stress-induced senescence and its therapeutic targeting, several critical gaps must be addressed to ensure successful clinical translation.

One promising area lies in the identification and validation of non-invasive biomarkers of senescence. Circulating microRNAs (miRNAs) have emerged as potential diagnostic and prognostic tools due to their stability in bodily fluids and their capacity to reflect intracellular signaling states. Specifically, miR-21 and miR-146a are consistently dysregulated in aging and senescence-associated diseases. miR-21, which modulates the p53 pathway, TGF-β signaling, and oxidative stress responses, is upregulated in various cancers and cardiovascular pathologies, whereas reduced levels have been associated with healthy aging. Similarly, miR-146a plays a pivotal role in dampening inflammatory signaling via the NF-κB axis, and its altered expression is linked to immune senescence and inflammaging. Both miRNAs show potential as dynamic biomarkers for monitoring senescence burden and therapeutic response. However, their implementation in clinical settings requires standardization of detection protocols, robust cohort-based validation, and longitudinal studies to establish temporal specificity.

Additionally, clinical and translational validation of senotherapeutic strategies remains an unmet need. While several senolytic and senomorphic compounds have shown promise in preclinical and early-phase clinical trials, the heterogeneity of senescent phenotypes across tissues and diseases demands the development of predictive preclinical models that more faithfully recapitulate human senescence. Three-dimensional organoids, patient-derived xenografts, and humanized mouse models offer opportunities for more relevant drug testing and biomarker discovery.

Equally critical is the standardization of clinical endpoints. Current trials often rely on surrogate markers, such as a reduction in SASP components or improvements in physical function, which may not capture the full spectrum of therapeutic effects. Future studies should incorporate composite outcomes encompassing functional, molecular, and quality-of-life indicators, along with validated biomarkers such as circulating miRNAs and imaging modalities capable of tracking senescent cell burden in vivo.

Moreover, advances in systems biology, artificial intelligence, and single-cell omics are poised to revolutionize the senescence field. These tools will enable the deconvolution of senescence heterogeneity, identification of senescence subtypes, and prediction of individual responses to senotherapeutics.

In conclusion, while targeting senescence offers transformative potential for age-related and degenerative diseases, its success will hinge upon integrative efforts that bridge mechanistic insight, biomarker development, and rigorous translational validation.

## Figures and Tables

**Table 1:** Overview of reactive oxygen species: types, sources, antioxidant defenses, and biological roles.

Aspect	Description	References
Definition of ROS	Chemically reactive molecules containing oxygen, including radical forms (e.g., superoxide, hydroxyl radical) and non-radical forms (e.g., hydrogen peroxide).	[4,5,6,7,8,9,10,11]
Types of ROS	- Highly reactive (hiROS): Hydroxyl radicals, singlet oxygen. - Less reactive (loROS): Superoxide, hydrogen peroxide.	[10]
Sources of ROS	- Endogenous: Mitochondrial respiration, NADPH oxidases, xanthine oxidase, nitric oxide synthases, cytochrome P450, peroxisomal β-oxidation. - Exogenous: Environmental pollutants, ionizing radiation, xenobiotics, pharmaceuticals, dietary factors, immune responses (via phagocytes).	[11,12,13,14,15,16,17,18,19,20,21,22,23,24]
Antioxidant Defenses	- Enzymatic: Superoxide dismutases catalase), glutathione peroxidases, glutathione reductase, thioredoxin reductases. - Non-enzymatic: Glutathione, vitamins C and E, uric acid, carotenoids. - Preventive antioxidants: Transferrin, ferritin, ceruloplasmin, albumin (metal chelators).	[25,26,27,28,29,30,31,32,33,34,35,36,37,38,39]
Key Regulatory Pathway	Keap1–Nrf2 pathway: Controls antioxidant gene expression in response to oxidative stress. Nrf2 activity declines with age, increasing susceptibility to oxidative damage.	[34,35,36,37]
Physiological Roles of ROS	Involved in cell signaling, transcriptional regulation, immune responses, and stem cell fate decisions; mild ROS levels contribute to mitohormesis and healthspan extension. Chronic ROS accumulation leads to oxidative stress, lipid/protein/DNA damage, genomic instability, chronic inflammation, metabolic dysfunction, and promotes tumorigenesis.	[3,31,47,48,49,50,51,52,53]

**Table 2:** Molecular triggers, features, and clinical implications of cellular senescence.

Trigger/Mechanism	Molecular Pathways Involved	Key Cellular Changes	Clinical Implications	References
Telomere Attrition	DDR activation via p53/p21	Cell cycle arrest, SAHF formation	Limits somatic cell renewal, contributes to aging-related tissue degeneration and reduced regenerative capacity	[66,67,68,69,70]
DNA Damage (e.g., radiation, chemotherapy)	ATM/CHK2/p53/p21 and ATR/CHK1/p38/p16 pathways	Growth arrest, DNA damage foci	Protects against tumorigenesis; excessive damage linked to therapy-induced senescence and secondary malignancies	[71,72,73,74]
Oxidative Stress and Mitochondrial Dysfunction	ROS production, p53 activation, metabolic reprogramming	Mitochondrial ROS accumulation, shift to glycolysis	Drives inflammaging, metabolic syndrome, cardiovascular diseases, and neurodegeneration	[62,75,76,77]
Metabolic Stress (e.g., glucose overload)	mTOR, AMPK signaling	Impaired mitochondrial respiration, lipid metabolism disruption	Associated with obesity, diabetes, and premature aging	[62,78,79,80,81,82]
Endoplasmic Reticulum (ER) Stress	UPR activation (PERK, ATF6, IRE1), p53/p21	Protein misfolding, impaired proteostasis	Contributes to neurodegenerative diseases and age-related organ dysfunction	[83,84,85,86,87]
Oncogene-Induced Senescence (OIS)	RAS/RAF/MEK, p16, ARF, p53 pathways	SASP production, stable growth arrest	Early tumor suppression; chronic OIS may promote tumor progression via SASP-mediated microenvironment changes	[88,89,90,91]
Epigenetic Remodeling	DNA hypomethylation, histone modifications (e.g., H3K4me3 loss)	Chromatin reorganization, SAHF formation	Epigenetic instability contributing to aging, cancer predisposition	[95,96,97]
Morphological and Functional Alterations	p53/p21 and p16/Rb axes; cytoskeletal reorganization	Cell enlargement, altered organelle function, increased SA-β-gal	Diagnostic markers of aging cells; target for senotherapeutic strategies	[2,66,98,103,104,105,106,107,108,109,110,111,112]
Senescence-Associated Secretory Phenotype (SASP)	NF-κB, C/EBPβ, cGAS–STING pathways	Secretion of cytokines, chemokines, growth factors, proteases	Promotes chronic inflammation, fibrosis, cancer progression, tissue repair, and immune clearance of damaged cells (data)	[2,60,117,118,119,120]

**Table 3:** Oxidative stress-driven mechanisms of cellular senescence and associated clinical implications.

Mechanism/Pathway	Molecular Events and Key Players	Cellular/Functional Outcome	Clinical Implications	References
DNA Damage Response (DDR) Activation	ROS-induced DNA lesions, especially at telomeres; ATM/ATR, GADD45–p38MAPK–GRB2–TGF-β axis	Persistent DDR foci, growth arrest, SAHF	Promotes genomic instability; contributes to cancer, aging, and degenerative diseases	[31,121,122,123,124,125,126,127,140,141,145]
Mitochondrial Dysfunction	mtDNA damage, impaired oxidative phosphorylation, ROS amplification	Vicious cycle of ROS production, metabolic reprogramming, SASP activation	Inflammaging; metabolic disorders; neurodegeneration; mitochondrial diseases	[131,132,133,163,164,165,166]
AMPK Activation and Metabolic Checkpoints	AMPK stabilization of p21/p16 mRNAs, redox-sensitive checkpoints	Reinforced senescence via p53/Rb pathways	Potential target for metabolic syndrome and age-related disease intervention	[137,138,139]
TSP1–CD47–Nox1 Axis	Thrombospondin-1 activation of NADPH oxidase (Nox1), ROS production	Amplified p53 signaling, DNA damage response	Implicated in vascular aging, atherosclerosis, and tumor progression	[156,157,158,159,160]
Nrf2–mTOR Axis	Nrf2 antioxidant response, Nrf2-mTOR reciprocal regulation	Balances SASP, regulates immune clearance vs. persistence of senescent cells	Target for therapies in aging, cancer, and chronic inflammation	[2,78,90,171,172,173,174,175,176,177,178,179,180]
Oxidative Stress-Induced SASP Amplification	NF-κB, C/EBPβ activation by ROS, SASP gene upregulation	Proinflammatory secretome, paracrine senescence	Drives chronic inflammation, tissue degeneration, and cancer progression	[2,91,167,168,169,170]
MicroRNAs in Oxidative Senescence	miR-21, miR-34a, miR-146a, miR-210, miR-494; ROS-modulated expression	Modulation of redox balance, autophagy inhibition, mitochondrial dysfunction	Biomarkers for aging and age-related diseases; potential therapeutic targets	[3,183,184,185,186,187,188,189,190,191,192]
Telomere–Mitochondria Crosstalk	Mitochondrial ROS induce telomere shortening; telomere dysfunction impairs mitochondria	Systemic redox imbalance, cellular dysfunction	Accelerated aging; cardiovascular and neurodegenerative diseases	[3,141,142,143]

**Table 4:** Senotherapeutic strategies, mechanisms of action, and clinical implications.

Senotherapeutic Approach	Mechanism of Action	Key Agents/Strategies	Clinical Implications	References
Senolytics	Selective apoptosis of senescent cells by inhibiting pro-survival pathways (includes agents with overlapping antioxidant functions, e.g., flavonoids)	Dasatinib + Quercetin (D+Q), Navitoclax (ABT-263), Fisetin, FOXO4-DRI peptide, HSP90 inhibitors	Alleviates age-related diseases (e.g., idiopathic pulmonary fibrosis, cardiovascular diseases, and cancer); risks include tissue regeneration impairment and thrombocytopenia	[193,195,196,197,198,199,200,201,202,203,204,205,206,208,209,210,211,212,217,218,219,220]
Senomorphics	Suppression or modulation of SASP without killing senescent cells	Rapamycin, JAK inhibitors (ruxolitinib, baricitinib), Metformin, Apigenin, Kaempferol, FK866, p38 MAPK inhibitors, BET inhibitors	Reduces chronic inflammation, improves tissue homeostasis, manages cancer, osteoarthritis, and neurodegeneration; essential to balance SASP suppression to preserve tissue repair	[193,221,222,223,224,225,226,227,228,229,230,231,232,233,234,235,236]
Redox Modulation	Modulates oxidative stress to influence senescence induction or suppression	Resveratrol, Ginsenoside Rh2, Apigenin, Phloretin, Genistein, Sulforaphane, Antioxidant mimetics, Nrf2 activators	Protects non-cancer tissues from oxidative stress; induces selective cancer cell senescence; potential in cancer therapy, aging, and metabolic diseases	[12,111,237,238,239,240,241,242,243,244,245,246,247,248,249,250,251]
Gene Therapy	Genetic modification to restore cell cycle regulators or eliminate senescent cells	CDK2/RB modulation, suicide gene therapy with senescence-specific promoters	Potential treatments for neurodegeneration, cataracts, and fibrosis; enhances precision in removing senescent cells while preserving healthy tissue	[62,251,252]
Immunotherapy	Enhancing immune clearance of senescent cells via engineered immune cells or checkpoint inhibition	CAR-T cells targeting uPAR, NK cell activation via NKG2D, PD-L1 checkpoint inhibitors, EZH2 inhibitors	Promising for cancer therapy, immune rejuvenation in aging, and reducing senescence burden in age-related diseases	[253,254,255,256,257,258,259,260,261,262]

## Data Availability

As this is a review article, no original research data were generated or analyzed. The manuscript provides a comprehensive synthesis and critical appraisal of previously published studies, all of which are appropriately cited. Consequently, a data availability statement is not applicable.

## Abbreviations

The following abbreviations are used in this manuscript:

AGO2	Argonaute 2
ALDH4A1	aldehyde dehydrogenase 4 family member A1
AMPK	AMP-activated protein kinase
AP-1	activator protein 1
AREs	antioxidant response elements
ARF	alternate reading frame
ATR	Rad3-related
ATM	ataxia-telangiectasia mutated
ATF6	activating transcription factor 6
Akt	protein kinase B
BAX	Bcl-2-associated X protein
BCL-2	B-cell lymphoma 2
BCL-XL	B-cell lymphoma-extra large
BET	bromodomain and extraterminal domain
CAR	chimeric antigen receptor
CAT	catalase
CCL2	C-C motif chemokine ligand 2
CD47	cluster of differentiation 47
CDK	cyclin-dependent kinase
CDKN1A	cyclin-dependent kinase inhibitor 1A
cGAS–STING	GMP-AMP synthase–stimulator of interferon genes
CHK2	checkpoint kinase 2
CHOP	C/EBP homologous protein
C/EBPβ	CCAAT/enhancer-binding protein beta
D	dasatinib
DDR	DNA damage response
DNA	deoxyribonucleic acid
DRI	D-retro-inverso
EMT	epithelial–mesenchymal transition
ER	endoplasmic reticulum
ETC	electron transport chain
EZH2	enhancer of zeste homolog 2
FOXO	forkhead box O
FOXO4	forkhead box O4
GADD45A	growth arrest and DNA damage-inducible alpha
GGT	glutamyl transpeptidase
GLS2	glutaminase 2
GR	glutathione reductase
GRB2	growth factor receptor-bound protein 2
H3K4me3	histone 3 lysine 4 trimethylation
HIF-1α	hypoxia-inducible factor 1-alpha
HO-1	heme oxygenase-1
HSP90	heat shock protein 90
HuR	human antigen R
IFN-γ	interferon-gamma
IL	interleukin
IRE1	inositol-requiring enzyme 1
JAK	Janus kinase
Keap1	Kelch-like ECH-associated protein 1
loROS	less reactive ROS
Maf	musculoaponeurotic fibrosarcoma
MAPK14	mitogen-activated protein kinase 14
MEK	mitogen-activated protein kinase kinase
miRNAs	microRNAs
MKK3	mitogen-activated protein kinase kinase 3
MMP1	matrix metalloproteinase 1
MMP3	matrix metalloproteinase 3
MnSOD	manganese superoxide dismutase
mTOR	mechanistic target of rapamycin
mTORC1	mechanistic target of rapamycin complex 1
MSC	mesenchymal stem cells
mtDNA	mitochondrial DNA
NAMPT	nicotinamide phosphoribosyltransferase
NAD^+^	nicotinamide adenine dinucleotide
NADPH	nicotinamide adenine dinucleotide phosphate
NF-κB	nuclear factor kappa-light-chain-enhancer of activated B cells
NISP	Nrf2-induced secretory phenotype
NK	natural killer
NKG2D	natural killer group 2 member D
Nox1	NADPH oxidase 1
Nox2	NADPH oxidase 2
NQO1	NAD(P)H quinone oxidoreductase 1
Nrf2	nuclear factor erythroid 2–related factor 2
O-GlcNAcylation	O-linked N-acetylglucosamine glycosylation
OIS	oncogene-induced senescence
PDGF-AA	platelet-derived growth factor-AA
PD-L1	programmed death-ligand 1
PERK	protein kinase RNA-like endoplasmic reticulum kinase
PGC-1β	peroxisome proliferator-activated receptor gamma coactivator 1-beta
PI3K	phosphoinositide 3-kinase
PIG1–13	p53-induced genes 1–13
p53	tumor protein p53
pRB	retinoblastoma protein
PS-NPs	polystyrene nanoplastics
PUMA	p53 upregulated modulator of apoptosis
Q	quercetin
RAF	rapidly accelerated fibrosarcoma
RAS	rat sarcoma viral oncogene homolog
RNS	reactive nitrogen species
ROS	reactive oxygen species
SA-β-gal	senescence-associated β-galactosidase
SAHF	senescence-associated heterochromatin foci
SASP	senescence-associated secretory phenotype
SIPS	stress-induced premature senescence
SNCs	senescent cells
SODs	superoxide dismutases
STAT	signal transducer and activator of transcription
TERT	telomerase reverse transcriptase
TGF-β	transforming growth factor-beta
TIGAR	TP53-induced glycolysis and apoptosis regulator
TIS	therapy-induced senescent
TNF-α	tumor necrosis factor-alpha
TSP1	thrombospondin-1
uPAR	urokinase-type plasminogen activator receptor
UPR	unfolded protein response
UV	ultraviolet
